# DOE-LVI: Tightly Coupled LiDAR-Visual-Inertial SLAM System with Dynamic Object Elimination

**DOI:** 10.3390/s26123717

**Published:** 2026-06-11

**Authors:** Tuanjie Li, Shichao Yang, Xu Li, Junjie Wang

**Affiliations:** 1 College of Information Engineering, Tarim University, Alar City 843300, China; 2 School of Mechano-Electronic Engineering, Xidian University, Xi’an 710071, China; 3 Baidu Inc., Beijing 100193, China

**Keywords:** dynamic environment, LiDAR-visual-inertial odometry, place recognition, simultaneous localization and mapping

## Abstract

In dynamic environments, Simultaneous Localization and Mapping (SLAM) systems often struggle with the challenges posed by moving objects. To address these issues, we propose Dynamic-Object-Elimination LiDAR-Visual-Inertial SLAM (DOE-LVI), an advanced tightly coupled LiDAR-Visual-Inertial SLAM system. DOE-LVI integrates two primary subsystems: the Visual-Inertial System (VIS) and the LiDAR-Inertial System (LIS). The VIS component extracts depth information from LiDAR scans and correlates it with visual features, providing accurate pose estimation by minimizing both visual and IMU residuals. The LIS uses this initial estimate to generate range images and perform preliminary removal of dynamic points. Misclassified points are then corrected through ground fitting and precise scan matching with the submap. For enhanced loop closure detection, DOE-LVI employs global LiDAR descriptors, which significantly improve both localization robustness and accuracy. Experimental evaluations on the KITTI and UrbanNav datasets demonstrate that DOE-LVI achieves robust localization and mapping performance, particularly in highly dynamic environments.

## 1. Introduction

Simultaneous Localization and Mapping (SLAM) is a fundamental capability for mobile robot navigation. By leveraging the unique characteristics of different sensors and fusing their data, SLAM systems can overcome the limitations of individual sensors, thereby improving accuracy, robustness, and applicability in complex environments [1,2,3]. For instance, LiDAR Odometry and Mapping (LOAM) [4] employs a loosely coupled approach, utilizing a Kalman filter to fuse LiDAR data with IMU measurements. High-frequency motion data from the IMU are utilized to correct the point cloud, enabling precise pose estimation. LiDAR-Inertial Odometry via Smoothing and Mapping (LIO-SAM) [5] is a tightly coupled LiDAR-Inertial Odometry system that leverages IMU pre-integration to compensate for point cloud distortion and provide an initial estimate for scan matching, resulting in faster processing and improved trajectory accuracy. LiDAR-Visual-Inertial Odometry via Smoothing and Mapping (LVI-SAM) [6] extends LIO-SAM by integrating a Visual-Inertial System alongside the LiDAR-Inertial System, with both subsystems tightly coupled to achieve robust and high-precision state estimation and mapping. Representative LiDAR-Inertial-Visual fusion systems, such as R3LIVE [7], further demonstrate that integrating LiDAR, inertial, and visual measurements can improve the robustness and accuracy of real-time state estimation and mapping. However, the mainstream SLAM algorithms [5,6,7,8,9] are primarily designed for static environments. In real-world scenarios, a more common situation involves complex scenes composed of both dynamic objects and static structures. Due to sensor occlusions, many features become associated with moving objects, leading to the failure of static-scene-based methods.

The primary methods for dynamic object removal typically involve comparing the point clouds across both time and space. Recent LiDAR moving object segmentation methods further exploit sequential range images to distinguish moving and static objects in 3D LiDAR scans [10]. For example, Kim et al. [11] utilized known global poses to construct a submap for each LiDAR scan and extracted dynamic point sets using a multi-resolution range image. Lim et al. [12] extracted descriptors from LiDAR scans and submaps, marking regions with low descriptor ratios as dynamic, where non-ground points were labeled as dynamic objects. However, these methods depend heavily on accurate global poses, and in environments with numerous dynamic objects, existing scan-matching techniques often struggle to provide the required accuracy. This limitation reduces the effectiveness of these methods for online use in dynamic environments. In contrast, Removal-First LiDAR-Inertial Odometry (RF-LIO) [13] offers an online solution by using adaptive multi-resolution range images to remove dynamic objects, followed by scan matching, enabling real-time operation in dynamic scenarios. Wen Lim et al. [14] proposed a label consistency-based dynamic point removal method, which reduces computational overhead and enables online localization and mapping.

In addition, loop closure is a critical step in SLAM, essential for correcting odometry drift and creating a globally consistent map. Most LiDAR-based loop closure detection methods rely on odometry. They use a k-dimensional tree (KD-tree) to find the closest keyframe in historical data as a loop closure candidate, then apply the Iterative Closest Point (ICP) algorithm to refine pose estimation. However, this method heavily relies on the system’s intrinsic accuracy and is significantly affected by odometry drift, leading to potential false loop closure candidates. In contrast, descriptor-based methods can mitigate the impact of accumulated errors. Scan-Context [15] encodes each LiDAR scan by representing the maximum height histogram in each bin of the horizontal plane as a point cloud descriptor. More recent structural place recognition methods, such as Scan Context++ [16], improve the robustness of LiDAR-based place recognition under rotation and lateral variations in urban environments. LiDAR-IRIS [17] further improved upon Scan-Context by using an 8-bit binary code to encode height information in each bin, providing rotation invariance and enhancing computational efficiency by avoiding brute-force matching.

In this work, we propose DOE-LVI, a tightly coupled LiDAR-Visual-Inertial Odometry system with dynamic object elimination, designed for real-time state estimation and mapping in long-term dynamic environments. DOE-LVI integrates a Visual-Inertial System (VIS) and a LiDAR-Inertial System (LIS). The VIS tracks visual features and estimates the pose by minimizing visual reprojection errors and IMU residuals. An active failure detection module assesses sensor reliability, providing the appropriate initial guess for LiDAR scan matching. The LIS first performs a coarse removal of dynamic points using range images between the current scan and the surrounding submap, then refines this process by recovering incorrectly identified points through ground plane fitting, followed by accurate pose estimation via scan matching. For loop closure detection, the LIS employs a global descriptor-based [17] method for place recognition, which is resilient to the influence of odometry drift. Compared with LIO-SAM and LVI-SAM, DOE-LVI is specifically designed for highly dynamic environments by introducing online dynamic object elimination before LiDAR scan matching. Unlike ERASOR, which relies on a pre-built static map, DOE-LVI removes dynamic points online by using the current scan and the surrounding submap. Compared with RF-LIO, DOE-LVI further integrates visual-inertial constraints, active failure detection, and global descriptor-based loop closure detection within a tightly coupled LiDAR-Visual-Inertial framework. Therefore, the main novelty of DOE-LVI lies in a dynamic environment-oriented system-level integration, together with an improved coarse-to-fine dynamic point removal strategy. It should be noted that LiDAR-IRIS is adopted as an existing global descriptor for loop closure detection, rather than being proposed as a new descriptor in this work. Our contribution lies in integrating it into the DOE-LVI framework to improve long-term mapping consistency in dynamic environments. The main contributions of our work can be summarized as follows:

(1) A dynamic environment-oriented, tightly coupled LiDAR-Visual-Inertial Odometry framework is proposed, integrating active failure detection, online dynamic object elimination, and an adopted LiDAR-IRIS-based place recognition module into a unified system.
(2) Real-time dynamic point removal with initial rough elimination and refinement through ground fitting for improved accuracy.
(3) Comprehensive validations against state-of-the-art methods across various scales and environments.

The remainder of this paper is organized as follows: a framework for the proposed system is presented in Section 2. Experimental results are given in Section 3, with conclusions in Section 4.

## 2. The DOE-LVI Framework

**Figure 1:**
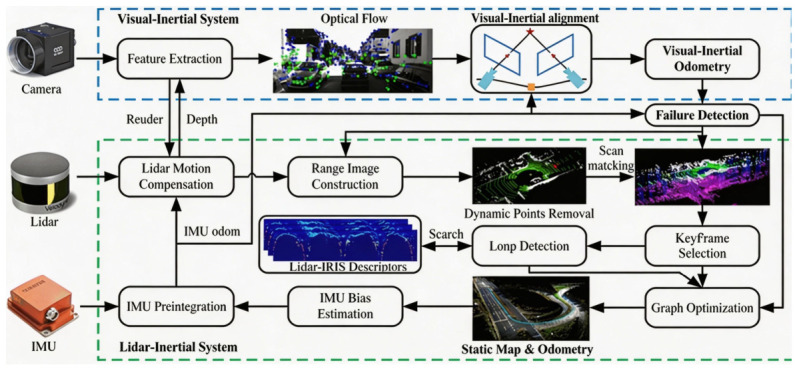
Overall framework of DOE-LVI.

Our framework consists of two key subsystems: a Visual-Inertial System and a LiDAR-Inertial System, as shown in Figure 1. The VIS renders the RGB color of the LiDAR scan, uses optical flow to track visual features, and obtains Visual-Inertial Odometry by optimizing errors of visual reprojection and IMU measurements. The LIS selects appropriate initial values based on the system state to construct a submap. It preliminarily removes dynamic points by comparing the range image of the scan and the corresponding submap and further refines dynamic points by fitting the ground. The static scan is then matched to the submap, and after graph optimization, a global static map and pose are obtained. We utilized LiDAR-IRIS descriptors [17] for pose recognition to eliminate cumulative errors. The overall framework of DOE-LVI is shown in Figure 1. The proposed system takes measurements from an IMU, a 3D LiDAR, and a camera as inputs, and mainly consists of a Visual-Inertial System (VIS) and a LiDAR-Inertial System (LIS). The VIS provides Visual-Inertial Odometry and evaluates its reliability through active failure detection, while the LIS performs dynamic object removal, scan matching, loop closure detection, and global map optimization. The optimized pose is further used to update the IMU bias, enabling the VIS and LIS to cooperate within the tightly coupled framework.

### 2.1. Visual-Inertial Odometry

**Figure 2:**
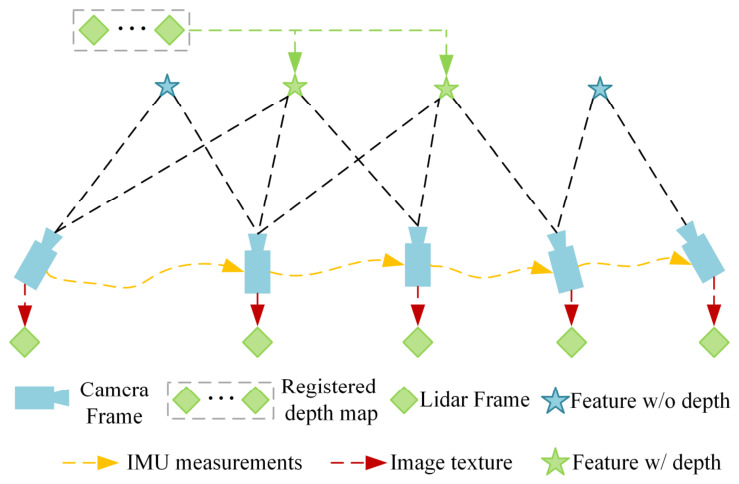
The framework of our Visual-Inertial System.

We have adapted and extended the processing pipeline from [18] for our VIS, as detailed in Figure 2. The VIS first utilizes the image information to render the texture of the LiDAR scan, then extracts key points and establishes accurate correspondences between adjacent image frames using the Kanade–Lucas–Tomasi algorithm. Finally, bundle adjustment optimization is performed within a sliding window. Note that the VIS does not include loop closure detection. Given that Visual-Inertial Odometry (VIO) can fail under aggressive motion, illumination changes, and textureless conditions, relying on VIO alone for initial estimates may introduce significant errors. Therefore, fault detection is crucial to mitigate adverse impacts on LiDAR-Inertial Odometry (LIO). Due to space constraints, this section emphasizes our modifications, particularly in LiDAR scan rendering and active failure detection. For further details on visual residuals and visual feature depth association, please refer to [6,18].

Rendering the texture of a LiDAR scan: Rendering helps in visually inspecting and verifying the quality of the point cloud data and its alignment. It allows for an intuitive understanding of the spatial distribution and structure of the point cloud. The motion-compensated LiDAR scan is projected onto the nearest image frame along the time axis and matched with the corresponding pixels in the RGB image to generate a 3D colored point cloud. A LiDAR point in the LiDAR coordinate system is denoted as pL=[XL,YL,ZL]T, and its homogeneous form is denoted as p˜L=[XL,YL,ZL,1]T. By using the extrinsic transformation from the LiDAR coordinate system to the camera coordinate system, the point is projected onto the image plane as follows:

(1) pC=XCYCZC=RCLpL+tCL, λucvc1=KRCLtCLLp˜, λ=ZC

where K is the camera intrinsic matrix, $R_{CL}$ and $t_{CL}$ denote the rotation matrix and translation vector from the LiDAR coordinate system to the camera coordinate system, respectively, and λ is the depth of the transformed point in the camera coordinate system. After normalization by $Z_{C}$, the pixel coordinates on the image plane are obtained as:

(2) $$u_{c}=f_{x}\frac{X_{C}}{Z_{C}}+c_{x}\;\mathrm{and}\;v_{c}=f_{y}\frac{Y_{C}}{Z_{C}}+c_{y}$$

where ${(u}_{c}{,v}_{c})$ denotes the pixel coordinates on the image plane. The RGB values are then extracted from the color image and assigned to the corresponding 3D LiDAR points to obtain the colored LiDAR scan.

Active Failure detection: In scenarios involving rapid vehicle motion or textureless environments, visual feature tracking frequently fails, hindering the convergence of optimization. To address this, when the IMU bias $b_{a}$, $b_{g}$, velocity $v_{imu}$, and translation $\Delta t_{s}$ between adjacent frames exceed predefined thresholds, the VIS reinitializes and informs the LIS. Here, $b_{a}$ and $b_{g}$ denote the accelerometer bias and gyroscope bias, respectively; $v_{imu}$ denotes the IMU-derived velocity; and $\Delta t_{s}$ denotes the translation between two adjacent frames. The threshold values of these parameters are listed in Table 1. These thresholds were selected empirically according to the IMU noise characteristics and preliminary experiments. Once determined, they were kept fixed across all datasets and sequences, including KITTI, Semantic-KITTI, and UrbanNav, rather than being tuned separately for each sequence. The purpose of these thresholds is to conservatively detect abnormal VIS behavior and prevent unreliable VIO constraints from being added to the factor graph. It should be noted that these thresholds are not intended to be universal constants for all sensor platforms. For systems with different IMU noise levels, sensor configurations, motion characteristics, or operating environments, these thresholds may need to be recalibrated through preliminary experiments.

We observed that unstable VIS outputs can significantly affect the overall system performance. During the short recovery period after a VIS reboot, stable VIO constraints are not yet available. Therefore, VIO constraints are temporarily excluded from the factor graph to avoid degrading LiDAR-Inertial optimization. Instead, the initial value of the current frame is estimated using the optimized pose from the previous frame and IMU propagation. Then, point cloud registration is performed, and the current frame pose is obtained through factor graph optimization. The optimized LIO result is further used to update the IMU bias. In this way, active failure detection improves the stability of the system in complex scenarios.

### 2.2. Dynamic Point Removal

Dynamic point removal requires comparison of point clouds across different frames [9]. For each point cloud frame containing dynamic objects, a submap is constructed using nearby multi-frame point clouds. The difference between the range image of the current point cloud frame and the submap is used to generate a residual image. This residual information is then employed to partition the target map into two mutually exclusive subsets: the static point cloud map and the dynamic point cloud map.

**Figure 3:**
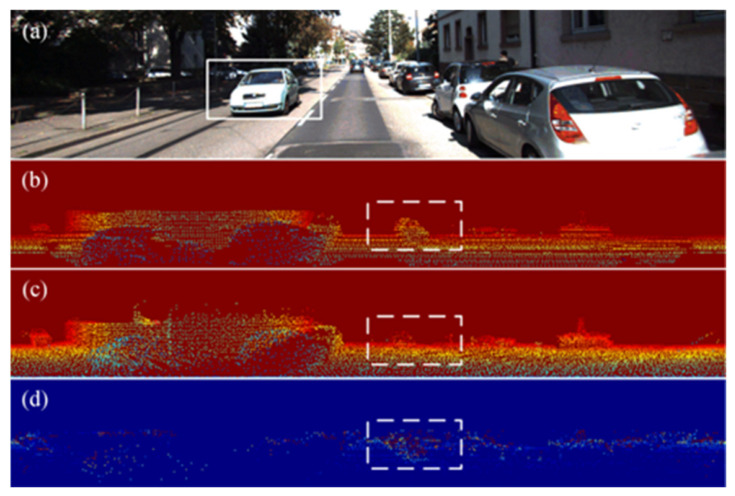
Visibility-based dynamic point removal. (**a**) a scene containing dynamic objects. (**b**) the range images generated from the current scan. (**c**) the range images generated from he surrounding submap. (**d**) a residual imag by comparing the two range images.

The visibility-based dynamic point removal process is shown in Figure 3. Figure 3a presents a scene containing dynamic objects. Figure 3b,c show the range images generated from the current scan and the surrounding submap, respectively. By comparing these two range images, a residual image is obtained, as shown in Figure 3d. Pixels with large residual values indicate inconsistent observations between the current scan and the submap, and these points are identified as potential dynamic points.

**Figure 4:**
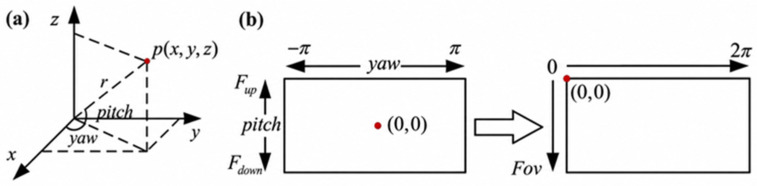
Range image construction. (**a**) LiDAR point transformation. (**b**) the full 3D LiDAR scan is unfolded into a 2D range image.

Dynamic point filter: The construction process of the range image is illustrated in Figure 4. First, each LiDAR point is transformed from the Cartesian coordinate system to the spherical coordinate system, as shown in Figure 4a. Then, the full 3D LiDAR scan is unfolded into a 2D range image according to the azimuth and elevation angles, as shown in Figure 4b.

The vertical field of view of the LiDAR is divided into an upper part $F_{up}$ and a lower part $F_{down}$. The laser point $p\left(x,y,z\right)$ in the Cartesian coordinate system, as shown in Figure 4a, is represented in the spherical coordinate system as:

(3) $$\left\{\begin{array}{l} r=\sqrt{x^{2}+y^{2}+z^{2}}\\ \theta =yaw=-\arctan\left(y,x\right)\\ \varphi =pitch=\arcsin\left(z/r\right) \end{array}\right.$$

where *x*, *y*, and *z* are the coordinates of the LiDAR point in the LiDAR coordinate system; *r* denotes the range from the LiDAR origin to the point; *yaw* or *θ* denotes the horizontal azimuth angle; and *pitch* or *φ* denotes the vertical elevation angle.

After scanning a full circle with the LiDAR, the point cloud is unfolded into a 2D image, with the x direction as the front view. As shown in Figure 4b, the image center is set as the origin; the vertical coordinate is obtained from the projection of the pitch angle, and the horizontal coordinate is obtained from the projection of the yaw angle. To account for variations in the LiDAR field of view, horizontal resolution, and image size, both coordinates are normalized. The final coordinates $\left(u,v\right)$ of the laser point projected onto the range image are given by:

(4) $$\left(\begin{array}{c} u\\ v \end{array}\right)=\left(\begin{array}{c} \frac{1}{2}\left[1-\arctan\left(y,x\right)/\pi \right]w\\ \left[1-\left(\arcsin\left(z/r\right)+F_{up}\right)/Fov\right]h \end{array}\right)$$

where *u* and *v* denote the horizontal and vertical pixel coordinates of the projected point in the range image, respectively; *w* and *h* denote the width and height of the range image; $F_{up}$ and $F_{down}$ represent the upper and lower vertical fields of view of the LiDAR, respectively; and *Fov* denotes the total vertical field of view.

We compare the current scan $F_{k}$ with the corresponding submap $M_{k}$ to remove dynamic points. A submap refers to the set of keyframes adjacent to the current frame in the spatiotemporal dimension. We assemble frames with dynamic points removed into a global map. Each submap is constructed from the previous global map, so the frames used to build each submap have already undergone dynamic point removal.

The scan is divided into two mutually exclusive subsets: the static points set $F^{S}$ and the dynamic points set $F^{D}$.

(5) $$F=F^{S}+F^{D}$$

The construction of the range image projects the 3D point cloud onto a 2D image, and when multiple 3D points project onto the same 2D point, the minimum distance from the 3D point $p\in R^{3}$ to the current scan $F_{k}$ is selected as the pixel value on the range image:

(6) $$I_{k}^{M}\left(i,j\right)=\underset{p\in P_{ij}^{M}}{\min}\;dist\left(p\right),\;I_{k}^{F}\left(i,j\right)=\underset{p\in P_{ij}^{F}}{\min}\;dist\left(p\right)$$

where $P_{ij}^{M}$ and $P_{ij}^{F}$ represent the spherical coordinates of the points $P^{M}$ and $P^{F}$, respectively, *i* represents the azimuth angle, *j* represents the elevation angle, and $dist\left(\cdot \right)$ represents the distance of the 3D point to the local coordinate system of the scan. The visibility of points is computed by subtracting their matrices element-wise within the same fixed coordinate frame.

(7) $$I_{k}^{diff}=\left|I_{k}^{F}-I_{k}^{M}\right|$$

When the pixel value $I_{k}^{diff}$ corresponding to the point $p\in P_{ij}^{F}$ is greater than the threshold τ, the point is considered to be a dynamic point:

(8) $$F_{k}^{D}=\left\{\left.F_{k}\right|I_{k}^{diff}>\tau \right\},\;\tau =k\times dist\left(p\right)$$

where *k* represents the sensitivity coefficient related to the point distance. The disparity in density between the scan and the submap, as observed in Figure 3, has resulted in the presence of numerous static points in the residual image, especially on the ground surface. This challenge can be mitigated by implementing an adaptive threshold and incorporating ground plane fitting methods.

Ground Plane Fitting: In real-world scenarios, most dynamic objects, such as moving vehicles and pedestrians, are above the ground. By classifying laser points as ground or non-ground, we can recover the ground points from the dynamic point set and add them to the static point set. For a scan *L*, the points are sorted by height, and the $n_{0}$ lowest points are selected to calculate the ground threshold. Based on this threshold, the initial set $S_{0}^{c}$ of candidate ground points is extracted as follows:

(9) $$S_{0}^{c}=\left\{\left.p_{k}\right|z\left(p_{k}\right)<\bar{z}+T_{init},p_{k}\in L,k\in \left(1,n_{0}\right)\right\}$$

where $z\left(p_{k}\right)$ represents the height value of point $p_{k}$, $\bar{z}$ is the average value of the $n_{0}$ lowest points, and $T_{init}$ is the initial ground candidate point height threshold. Based on the initial set of candidate points, an iterative plane optimization is performed to re-extract the optimized ground points. After *i* iterations, the covariance matrix $C_{i}$ of the ground points is obtained, and the eigenvalues and eigenvectors of the ground points in each direction through Principal Component Analysis (PCA) are obtained:

(10) Ci=∑k=1:Sicpk−p¯ipk−p¯iT

(11) $$C_{i}\cdot v_{j}=\lambda_{j}\cdot v_{j},j\in \left\{0,1,2\right\}$$

where $\left|S_{i}^{c}\right|$ represents the size of the set $S_{i}^{c}$, p¯i is the average position of all points in the candidate point set at the $i^{th}$ iteration, λ represents the eigenvalues of the covariance matrix, and v represents the corresponding eigenvectors.

According to PCA, the eigenvector corresponding to the smallest eigenvalue of the covariance matrix can represent the normal vector of the plane [19]. Let the normal vector be ni=ai,bi,ciT; the equation of the ground plane can be expressed as:

(12) aix+biy+ciz+di=0

Based on the plane equation, the plane coefficient $d^{i}=-{n^{i}}^{T}\bar{p}$. For each point $p_{L}$ in the point cloud frame, we define $d_{L}^{i}=-{n^{i}}^{T}p_{L}$. Then, the orthogonal point-to-plane distance from $p_{L}$ to the fitted plane is calculated as:

(13) $$D_{L}^{i}=\frac{\left|d^{i}-d_{L}^{i}\right|}{{\left\|n^{i}\right\|}_{2}},\;p_{L}\in L$$

When $D_{L}^{i}$ is less than the distance threshold $T_{\mathrm{dist}}$, the point is considered a ground point. Therefore, the ground point set in the (i+1)-th iteration is updated as:

(14) $$S_{i+1}^{c}=\left\{p_{L}\in L|D_{L}^{i}<T_{dist}\right\}$$

where $D_{L}^{i}$ denotes the orthogonal point-to-plane distance from point $p_{L}$ to the fitted ground plane, $n^{i}$ is the normal vector of the fitted plane, $d^{i}$ is the plane coefficient, $d_{L}^{i}$ is the projection coefficient of point $p_{L}$, and $T_{\mathrm{dist}}$ is the distance threshold for ground point extraction. This process iteratively optimizes the fitted ground plane and extracts the ground point set. After *n* iterations, the ground point set $S_{n}^{c}$ and the non-ground point set ${\bar{S}}_{n}^{c}=L-S_{n}^{c}$ are obtained. The portion of the dynamic points that belong to the ground is recovered and integrated back into the static point set. In Equations (8)–(14), $S_{0}^{c}$ denotes the initial candidate ground point set, $n_{0}$ is the number of lowest points used to estimate the initial ground height, $C_{i}$ denotes the covariance matrix at the $i^{th}$ iteration, $n^{i}$ and $d^{i}$ denote the normal vector and offset of the fitted ground plane, and $T_{dist}$ is the distance threshold for ground point extraction.

It should be noted that the core goal of this ground fitting module is to reduce the false removal of static ground points, which is the main source of precision loss in existing dynamic removal methods. For most dynamic points near the ground, the plane distance threshold can help exclude them from the ground point set during the iterative fitting process, thereby reducing the risk of misclassification. The slight decrease in recall after adding the ground fitting module is an acceptable trade-off for a significant improvement in precision, which ensures that the LIS can obtain sufficient static constraints for scan matching, avoiding system failure caused by excessive removal of static points.

However, low-height moving objects close to the ground may still be partially confused with ground points during the ground plane fitting stage. Examples include scooters, bicycles, wheelchairs, shopping carts, baby strollers, small mobile robots, animals, and moving road debris. Although range-image-based dynamic point detection can identify many of these objects through temporal inconsistency between the current scan and the submap, some low-height dynamic points may be recovered if they are very close to the fitted ground plane. This remains a limitation of the current geometric ground recovery strategy.

It should also be noted that the ground plane fitting module is mainly used to recover misclassified static ground points rather than to explicitly model all possible dynamic objects. For aerial dynamic objects or elevated moving structures, such as drones or objects moving above the ground surface, the ground fitting process usually does not recover them as ground points because they are far from the fitted ground plane. However, their removal still depends on the range-image-based dynamic point detection stage. If such objects are small, distant, sparsely scanned, or only temporarily observed, they may be difficult to detect reliably. Therefore, the current method is more suitable for ground-vehicle scenarios dominated by road users and ground structures, while aerial dynamic objects and elevated moving structures remain challenging cases for future work.

### 2.3. LiDAR-Inertial Odometry

Scan-Matching: Scan-matching is used to match the current scan $F_{k}$ with the surrounding submap $M_{k}$. In point cloud data, noise and outliers can disrupt point-to-point matching results. To address this, we convert the problem into finding correspondence between points in one point cloud and planes in another. By optimizing the distances between points and planes for alignment, the registration algorithm leverages plane geometry to achieve more accurate results.

For a point $p_{i}^{F}$ in the $F_{k}$, the VIO or IMU odometry is first used as an initial guess to transform $p_{i}^{F}$ to the submap as $p_{i}^{M}$. Then, the nearest point $p_{m}^{M}$ is found on the submap. Two additional points $p_{j}^{M}$ and $p_{k}^{M}$ are found from the laser beam containing $p_{m}^{M}$ and the surrounding beams. The three points on the submap are used to construct a plane $M_{mjk}$ [4].

**Figure 5:**
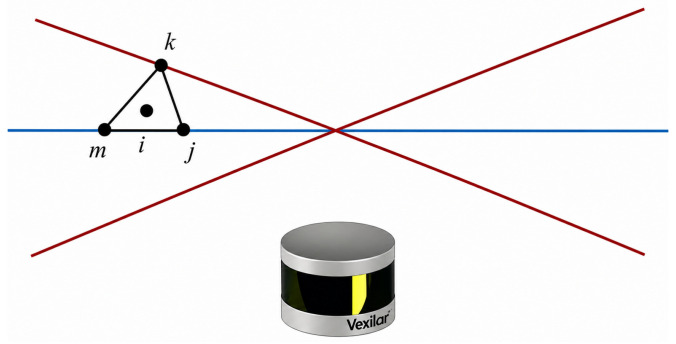
Local planar patch for scan matching.

The local planar patch used for scan matching is illustrated in Figure 5. For a feature point in the current scan, the nearest point is first searched in the surrounding submap. Then, two additional neighboring points are selected from the same laser beam and adjacent laser beams. These three points are used to construct a local plane, which converts scan matching into a point-to-plane distance minimization problem.

Then, the point-to-plane distance $d_{sm}$ is calculated as follows:

(15) $$n_{mjk}=\left(p_{m}^{M}-p_{j}^{M}\right)\times \left(p_{m}^{M}-p_{k}^{M}\right),n_{mjk}^{\prime }=n_{mjk}/\left|n_{mjk}\right|$$

(16) $$d_{sm}=\left|\left(p_{i}^{M}-p_{m}^{M}\right)\cdot n_{mjk}^{\prime }\right|$$

**Figure 6:**
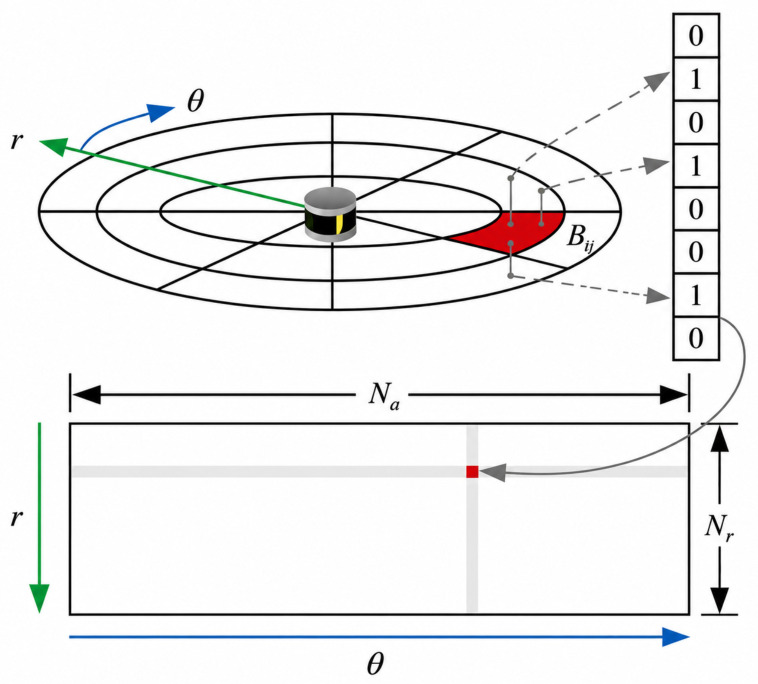
Encoding the LiDAR scan into the LiDAR-IRIS.

Loop Closure: This work adopts the global LiDAR descriptor LiDAR-IRIS [17] for place recognition. The encoding process of the LiDAR scan into the LiDAR-IRIS descriptor is shown in Figure 6. Each LiDAR scan is first divided into several bins. The height information within each bin is then encoded as an 8-bit binary code and converted into a decimal value, which is used as the pixel intensity in the LiDAR-IRIS image representation.

The LiDAR-IRIS image representation is formulated as follows: $N_{r}\times N_{a}$ bins. The height information within each bin is then encoded as an 8-bit binary code and converted to a decimal value, which is used as the pixel intensity in the LiDAR-IRIS image representation:

(17) $$I=\left(a_{ij}\right)\in R^{N_{r}\times N_{a}},a_{ij}=\phi \left(B_{ij}\right)$$

where $\phi \left(B_{ij}\right)$ represents the decimal encoding of the bin $B_{ij}$. The distance between the query frame and the candidate frame is computed using the Hamming distance. Specifically, for two binary feature images $b_{ij}^{q}$ and $b_{ij}^{c}$, the distance between the two LiDAR scans is given by:

(18) $$d=\sum_{i=1}^{N_{r}}\sum_{j=1}^{N_{a}}b_{ij}^{q}⊕b_{ij}^{c}$$

where ⊕ represents the XOR operation. We construct a row key KD-tree to speed up the loop closure candidate search. The binary features are re-encoded as an $N_{r}\times 1$ dimensional vector *K*:

(19) $$K=\left(\varphi \left(r_{1}\right),\cdots ,\varphi \left(r_{N_{r}}\right)\right),\;\varphi \left(r_{i}\right)=\frac{{\left\|r_{i}\right\|}_{2}}{N_{a}}$$

where $r_{i}$ represents the row vector of the feature image, and ${\left\|r_{i}\right\|}_{2}$ denotes its $L_{2}$ norm. For each scan, we construct a KD-tree using the *K* vector as the key, and then use the KD-tree to retrieve the top $N_{l}=10$ candidates from the keyframe database, selecting the frame with the smallest Hamming distance as the loop closure candidate. Candidate matches are validated by verifying feature correspondences between the scan and the surrounding submap using Random Sample Consensus (RANSAC). If the set of inliers is sufficiently large, the loop closure is deemed successful.

It should be noted that the LiDAR-IRIS-based loop closure detection operates mainly within the LIS and relies on LiDAR scan descriptors rather than visual features. Therefore, when the VIS is in a failed or rebooting state, the descriptor extraction and place recognition process can still be performed using LiDAR scans. During this period, the system uses the previous optimized pose and IMU propagation to provide the initial guess for LIO, while unreliable VIO constraints are not added to the factor graph. As a result, VIS failure does not directly interrupt LiDAR-IRIS-based place recognition. However, if the VIS remains unstable for a long time and LiDAR-Inertial Odometry accumulates large drift, the geometric verification of loop closure candidates may become more difficult. In such cases, successful loop closure can help correct accumulated drift once reliable LiDAR-based candidate matching is established.

**Figure 7:**
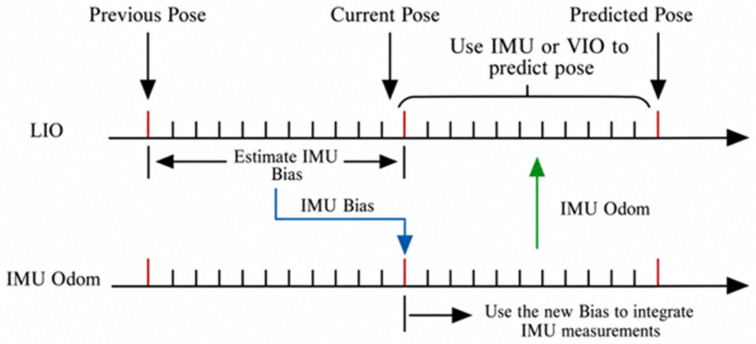
Estimation of IMU bias.

IMU Bias Estimation: When using IMU measurements for pose prediction, due to the time-varying IMU bias, it is necessary to update the IMU bias of the previous time step in real-time using the information from the LiDAR-Inertial Odometry. As shown in Figure 7, the current LiDAR scan pose is extracted from the factor graph optimization, and the IMU pre-integration quantities are calculated between the previous and current scans. Then, the IMU pre-integration factor, IMU bias factor, and pose factor are added to the factor graph for optimization, updating the current scan’s pose, velocity, and IMU bias. The latest bias is used to calculate the IMU pre-integration quantities for the next scan, predicting the next pose and publishing the IMU odometry.

## 3. Experiments

We compared the proposed framework with other state-of-the-art methods on the KITTI [20] and UrbanNav [21] datasets and evaluated the precision and recall of the dynamic point detection and removal method on Semantic-KITTI [22]. The KITTI dataset consists of outdoor urban environment sequences captured using a Velodyne HDL-64 LiDAR (California, USA), while the UrbanNav dataset was captured in the urban areas of Hong Kong using a Velodyne HDL-32 LiDAR (California, USA). Semantic-KITTI provides point-wise annotations, and each LiDAR point has its unique semantic label. Detailed information about the datasets is provided in Table 2.

The datasets are categorized into low, medium, and high levels based on the number of consecutive dynamic frames: less than 150 for low, 150–300 for medium, and over 300 for high. We used the root mean square error of the translational Absolute Trajectory Error (ATE-RMSE) to evaluate localization accuracy:

(20) $$ATE_{RMSE}=\sqrt{\frac{1}{n}\sum_{i=1}^{n}{\left\|t_{i}^{est}-t_{i}^{gt}\right\|}_{2}^{2}}$$

where n is the total number of poses, $t_{i}^{est}$ denotes the estimated position vector at time i, $t_{i}^{gt}$ denotes the corresponding ground-truth position vector, and $\|\cdot {\|}_{2}$ represents the Euclidean norm. In this work, only the translational component of the pose error is evaluated. In all experiments, we use the same parameters shown in Table 1.

**Figure 8:**
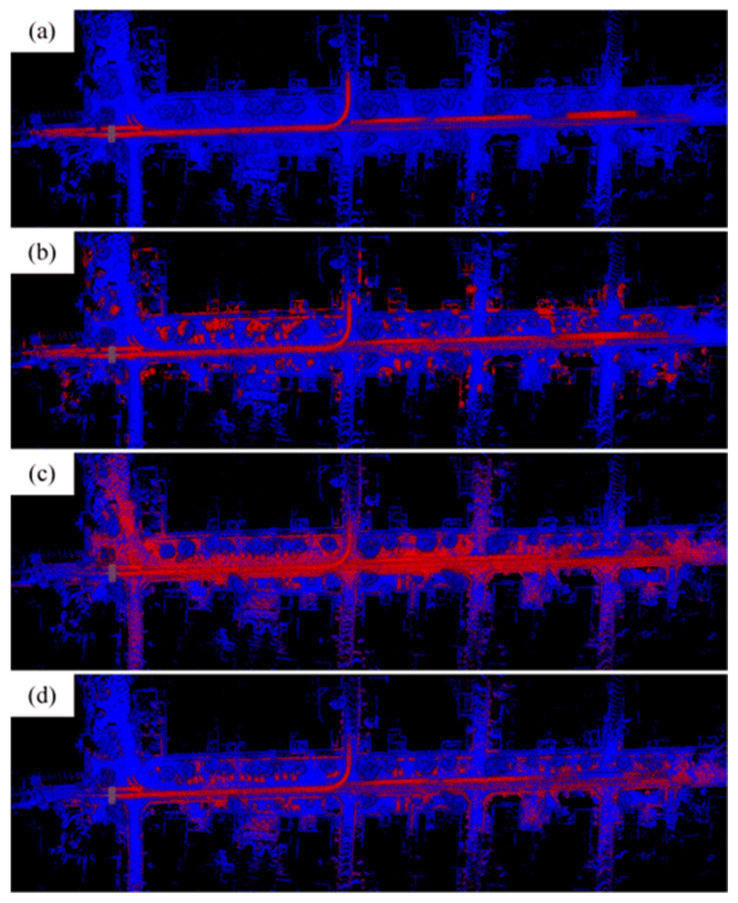
Comparison of static map generation results on sequence 05. (**a**) the ground truth. (**b**) the results of ERASOR. (**c**) the results of Ours without GPF. (**d**) the results of Ours.

Table 3 presents the precision (P), recall (R), and F1-score for all methods. ERASOR [12] is an offline method that requires a pre-built static map as input, whereas Ours and Ours (w/o GPF) are online methods that perform dynamic point removal using the current scan and the surrounding submap. Therefore, this comparison is mainly used to evaluate dynamic point removal behavior rather than to claim a strictly identical system-level setting. Ours (w/o GPF) refers to our dynamic point removal method without ground plane fitting to eliminate misclassified points. The results indicate that our method achieves a good balance between precision and recall, resulting in a higher F_1_-score. Although Erasor detected most of the dynamic points, it mistakenly removed a large portion of the static points, leading to the lowest precision. Similarly, Ours (w/o GPF) misclassified many static points on the ground, resulting in lower precision. Figure 8 shows the static map generation results of different methods on sequence 05. Figure 8a shows the ground truth, while Figure 8b–d present the results of ERASOR, Ours without GPF, and Ours, respectively. The red point clouds represent the dynamic points detected by each method.

We compared the proposed method with LIO-SAM [5] and LVI-SAM [6]. To demonstrate the effectiveness of dynamic point removal and LiDAR-IRIS, DOE-LVI-F and DOE-LVI-S represent our method without the dynamic point detection module and without LiDAR-IRIS, respectively. The results of all methods on the KITTI dataset are shown in Table 4.

It should be noted that ERASOR and our method are not compared under identical system assumptions. ERASOR uses an offline setting with a pre-built map, while our method performs online dynamic point removal. Therefore, the results in Table 3 should be interpreted as a comparison of dynamic point removal characteristics under different operating settings rather than as a direct comparison of complete SLAM systems under the same input conditions.

The results in Table 4 show that DOE-LVI achieves better accuracy than LIO-SAM and LVI-SAM on most KITTI sequences, especially in sequences with more dynamic objects or longer trajectories. However, on sequence 06, DOE-LVI performs slightly worse than LIO-SAM. Sequence 06 contains relatively few dynamic points and has a simple trajectory structure; therefore, the benefit of dynamic point removal and loop closure correction is limited. In such predominantly static scenarios, dynamic point removal may also remove a small number of useful static points or slightly perturb the scan-matching constraints, which can lead to a minor degradation in localization accuracy. Therefore, the result seen on sequence 06 indicates that the proposed dynamic point removal module is more beneficial in dynamic environments, while its advantage may be limited in nearly static scenes.

The ablation results also show that DOE-LVI-S performs worse than DOE-LVI-F on some sequences. Since DOE-LVI-F denotes the system without the dynamic point detection module and DOE-LVI-S denotes the system without the LiDAR-IRIS-based loop closure module, this result indicates that loop closure contributes more significantly than dynamic point removal in sequences where accumulated odometric drift is the dominant error source. Conversely, in highly dynamic sequences, dynamic point removal plays a more important role in improving scan-matching robustness. These results suggest that the relative contribution of each module depends on the dynamic level, trajectory length, and loop closure structure of each sequence.

**Figure 9:**
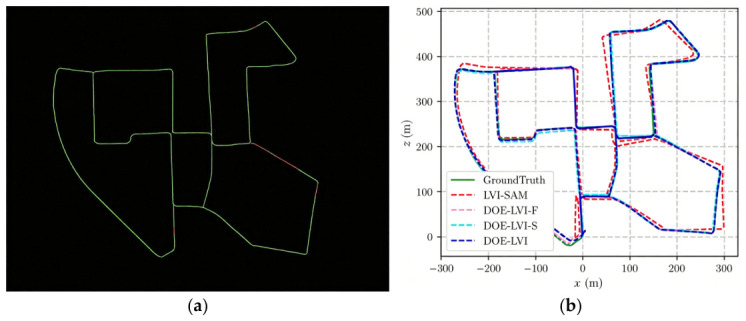
Trajectory comparison on KITTI sequence 00. (**a**) The detailed trajectory generated by DOE-LVI on sequence 00. (**b**) The trajectory comparison on sequence 00.

**Figure 10:**
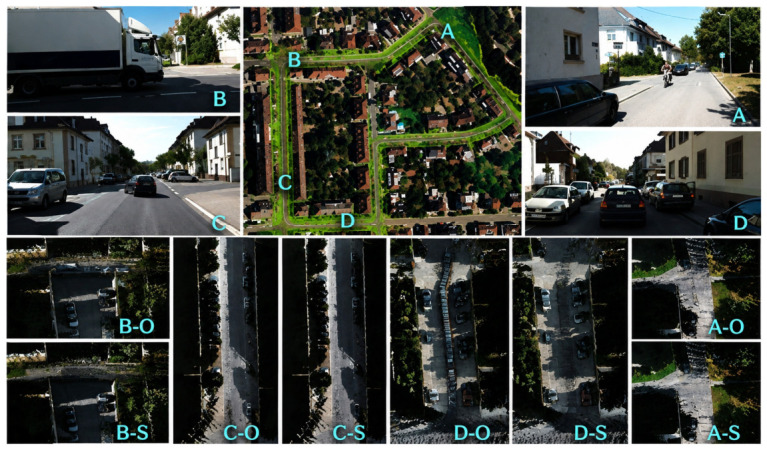
Mapping results on KITTI sequence 07.

The detailed trajectory generated by DOE-LVI on sequence 00 is shown in Figure 9a, where the green line represents the trajectory generated by the Visual-Inertial subsystem and the red line indicates the trajectory generated by the LIO subsystem after VIO tracking failure. Figure 9b presents the trajectory comparison on sequence 00. LVI-SAM is affected by VIO, resulting in larger errors in turns and on bumpy roads, whereas our method demonstrates better stability. Figure 10 presents the mapping result of DOE-LVI on KITTI sequence 07, including the map aligned with Google Earth and partial screenshots of dynamic objects. In Figure 10, “A-O” represents the original point cloud of location A, while “A-S” represents the static point cloud generated by DOE-LVI. The alignment with Google Earth indicates that the generated map is globally consistent.

**Figure 11:**
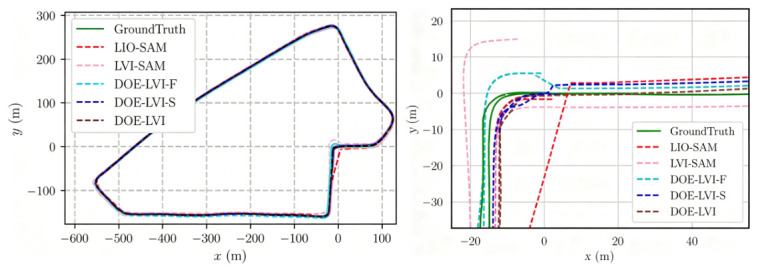
Trajectory comparison on 0428.

**Figure 12:**
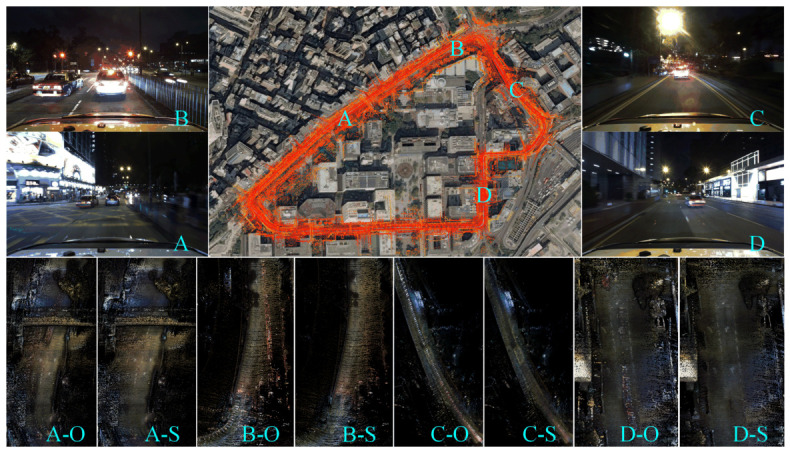
DOE-LVI map aligned with Google Earth and some dynamic objects on 0428.

To further evaluate the superiority of our method in challenging high-dynamic scenarios, we selected the UrbanNav dataset as the test dataset. The results of all methods on the UrbanNav dataset are shown in Table 5. LVI-SAM leverages visual odometry to provide an initial guess for LiDAR scan matching, hence resulting in inferior trajectory accuracy compared to LIO-SAM. Figure 11 illustrates the trajectory comparison on 0428. Figure 12 demonstrates the mapping result of DOE-LVI on UrbanNav 0428, showing high consistency between the generated map and Google Earth. In Figure 12, “A-O” represents the original point cloud of location A, while “A-S” represents the static point cloud generated by DOE-LVI.

The runtime experiments were conducted on a desktop computer equipped with an Intel Core i7 CPU, 16 GB RAM, and an NVIDIA GeForce RTX 3080 GPU, running Ubuntu 20.04. We compared the running time of DOE-LVI with other methods. The average processing time per frame across all datasets is shown in Table 6 for each module. The point cloud configuration parameters of LIO-SAM and LVI-SAM are set to their default values. The time consumption of our VIO subsystem is affected by image resolution, and the LIO is more significantly influenced by the density of the feature map. To demonstrate the point cloud rendering effect, we applied a lower downsampling rate. The time consumption of LIO is primarily concentrated in loop closure detection and backend optimization. In environments with rich features and a large number of dynamic objects, the time consumption of our dynamic points removal module will increase.

## 4. Conclusions

We have proposed DOE-LVI, a tightly coupled LiDAR-Visual-Inertial Odometry framework with dynamic object elimination for real-time and robust state estimation and mapping in highly dynamic environments. The proposed framework integrates the complementary advantages of visual, inertial, and LiDAR measurements, and improves the reliability of pose estimation through the cooperation between the Visual-Inertial System (VIS) and the LiDAR-Inertial System (LIS). DOE-LVI estimates the reliability of IMU odometry and VIO through active failure detection and determines whether IMU measurements or VIO results should be used as the initial guess for LIO. This strategy helps reduce the influence of unreliable visual constraints when visual tracking fails under rapid motion, illumination changes, or textureless scenes.

In the LIS, dynamic points are first removed before scan matching, which reduces the negative impact of moving objects on point cloud registration. To avoid excessive removal of useful static structures, ground plane fitting is further introduced to recover misclassified static ground points, thereby preserving sufficient constraints for accurate pose optimization. In addition, DOE-LVI employs a LiDAR-IRIS-based global descriptor for loop closure detection, which improves the system’s ability to correct accumulated drift and enhances the consistency of long-term localization and mapping. Through evaluations on datasets of various scales and environments, the results show that DOE-LVI achieves more stable localization performance in highly dynamic scenes. Compared with LVI-SAM, DOE-LVI reduces the ATE by 22% to 71% in highly dynamic environments, demonstrating the effectiveness of dynamic object elimination, active failure detection, and global descriptor-based loop closure in improving SLAM robustness.

In addition, the benefit of dynamic point removal may be limited in predominantly static environments, where unnecessary removal of a few static points may slightly affect scan-matching constraints. Nevertheless, low-height moving objects close to the ground may be partially confused with ground points during ground plane fitting, especially when their geometric height is close to the fitted ground plane. Moreover, aerial dynamic objects or elevated moving structures are not explicitly modeled in the current framework, and their removal mainly depends on the range-image-based dynamic point detection stage. In future work, we will optimize and improve the VIS to mitigate the influence of dynamic objects on vision and investigate semantic cues and temporal consistency constraints to better distinguish low-height dynamic objects, aerial dynamic objects, and elevated moving structures from static points.

## Figures and Tables

**Table 1:** Parameters used in DOE-LVI.

Parameters	$b_{a}$	$b_{g}$	$v_{imu}$	$\Delta t_{s}$	$n_{0}$	$T_{init}$	$T_{dist}$	k
Values	2.8	1.0	31.5	5.0	20	1.0	0.3	0.03

**Table 2:** Dataset details.

Dataset	Sequence	Scans	Trajectory Length (m)	Dynamic Level
KITTI	00	4541	3724.187	Low
KITTI	02	4661	5067.233	Low
KITTI	05	2761	2205.576	High
KITTI	06	1101	1232.876	Low88
KITTI	07	1101	694.697	Medium
KITTI	08	4071	3222.795	Medium
KITTI	09	1591	1705.051	High
KITTI	10	1201	919.518	Low
Urban	20190428	4871	1984.464	High
Urban	20200314	3005	1210.889	Medium
Urban	20210517	7848	3641.810	High

**Table 3:** Comparison of dynamic point removal on the Semantic-KITTI dataset.

	Method	P (%)	R (%)	$F_{1}$
00	ERASOR (offline)	4.250	93.200	0.081
	Ours (online, w/o GPF)	7.510	53.845	0.132
	Ours (online)	23.411	52.568	0.324
02	ERASOR (offline)	3.381	97.175	0.065
	Ours (online, w/o GPF)	7.412	59.514	0.132
	Ours (online)	17.623	53.675	0.265
05	ERASOR (offline)	10.690	91.275	0.191
	Ours (online, w/o GPF)	18.687	60.249	0.285
	Ours (online)	43.441	57.723	0.496
07	ERASOR (offline)	13.910	95.597	0.243
	Ours (online, w/o GPF)	33.795	48.319	0.398
	Ours (online)	50.274	46.944	0.486

**Table 4:** RMSE (m) comparison on the KITTI dataset.

Seq.	LIO- SAM	LVI- SAM	DOE- LVI-F	DOE- LVI-S	DOE- LVI
00	FAIL	11.30	3.46	4.10	3.25
02	4.55	FAIL	3.71	3.46	3.13
05	2.24	1.93	1.21	1.16	1.01
06	14.19	15.10	14.67	14.72	14.61
07	0.67	0.72	0.46	0.45	0.42
08	5.78	5.49	4.59	4.63	4.35
09	8.61	11.43	2.8	3.82	1.76
10	2.54	2.82	2.37	1.86	1.84

**Table 5:** RMSE (m) comparison on the UrbanNav dataset.

Seq.	LIO- SAM	LVI- SAM	DOE- LVI-F	DOE- LVI-S	DOE- LVI
0428	5.73	7.60	5.23	4.19	3.57
0314	1.74	2.51	1.83	1.95	1.42
0517	4.47	3.65	3.19	3.27	2.83

**Table 6:** Average processing time per frame for all methods.

Sequence	Image Size	LIO-SAM	LVI-SAM		DOE-LVI		
		LIO	VIO	LIO	VIO	LIO	Removal
00	1247 × 376	--	63.54	65.59	43.35	71.78	24.15
02	1241 × 376	66.89	--	--	45.47	68.45	22.89
05	1226 × 370	60.59	62.86	61.34	40.89	65.23	23.65
06	1226 × 370	89.34	54.63	71.68	37.88	64.01	20.51
07	1226 × 370	59.71	56.16	56.09	40.39	58.36	22.18
08	1226 × 370	60.25	68.25	64.24	43.56	62.45	23.76
09	1226 × 370	57.39	63.14	54.97	37.72	60.68	19.57
10	1226 × 370	52.69	59.18	56.78	36.21	55.46	18.67
0428	1920 × 1200	57.34	64.08	55.98	53.94	60.07	20.14
0314	1920 × 1200	51.21	75.06	54.21	57.67	61.41	19.80
0517	672 × 376	49.23	45.94	55.02	36.82	47.19	22.52

## Data Availability

Data are available on request from the authors.
